# Reverse Influence Modeling: Estimating Source-by-Source Health Benefits of Reduced Emissions

**DOI:** 10.1289/ehp.121-a166

**Published:** 2013-05-01

**Authors:** Lindsey Konkel

**Affiliations:** Lindsey Konkel is a Worcester, MA–based journalist who reports on science, health, and the environment. She writes frequently for *Environmental Health News* and *The Daily Climate*.

Traditional model-based studies that evaluate air quality control measures predict how the health benefits of a prescribed reduction in emissions will be distributed across different locations—for instance, how reformulating gasoline will benefit people in New York versus those in Los Angeles. But traditional models cannot feasibly quantify the influence of individual emissions sources on human health. Now researchers from Carleton University in Ontario have devised a method for tracing nationwide short-term mortality back to reductions at specific locations [*EHP* 121(5):572–579; http://dx.doi.org/10.1289/ehp.1205561].

The researchers integrated U.S. and Canadian epidemiological data with the adjoint, or reverse, of an air quality model based on a grid of cells measuring 36 square kilometers. This enabled them to estimate how reducing emissions within each grid cell would translate into public health benefits—i.e., decreased mortality. Although adjoint sensitivity models have been used in fields such as meteorology and atmospheric chemistry, this study is the first known to apply the method to epidemiological data derived from air quality time-series studies.

For Canada, the researchers calculated mortality from short-term exposure to ozone and nitrogen dioxide. For the United States, there was insufficient epidemiological evidence to link nitrogen dioxide to U.S. mortality, so they looked only at deaths in relation to ozone exposure.

**Figure:**
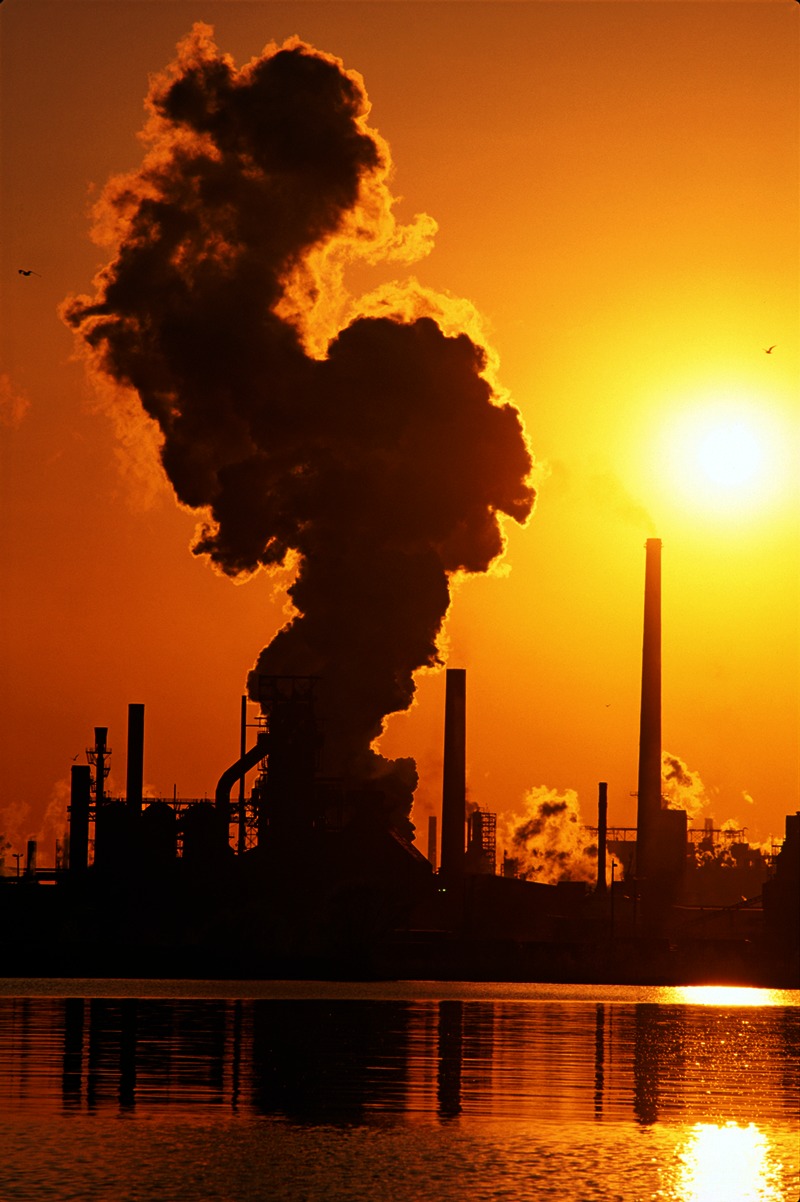
A Hamilton, Ontario, steel mill at sunset. © Cosmo Condina North America /Alamy

The authors found that the reductions in nitrogen dioxide and ozone exposure—and the consequent health benefits—associated with reducing nitrogen oxide emissions varied substantially across North America. For instance, a 10% reduction in emissions in Hamilton, Ontario, would result in estimated public health benefits from reduced mortality of Can$253,000 per day, while the same reduction in Detroit would benefit Canada by Can$47,000 per day. In the United States, a 10% reduction in nitrogen oxide emissions near Atlanta would result in estimated societal benefits of US$181,000 per day, whereas a 10% reduction in volatile organic compound emissions in New York would result in benefits of US$294,000 per day.

These figures represent only a fraction of the total expected health benefit. The researchers did not look at particulate matter, a known contributor to mortality. Nor did they consider long-term exposures to ozone or the societal costs associated with less fateful impacts of air pollution, such as asthma attacks or sick days.

In many cases, the researchers believe, the cost of polluting may be underestimated. In a benefit–cost analysis framework, reverse influence modeling may prove a useful tool in air quality decision-making by allowing for accurate estimates of the societal benefits of reducing emissions on a location-specific basis.

